# Incidence of Legg-Calvé-Perthes Disease in Crete (2021-2024): A Prospective Population-Based Epidemiological Study With Contemporary Literature Review

**DOI:** 10.7759/cureus.107954

**Published:** 2026-04-29

**Authors:** Emmanouela Dionysia Laskaratou, Babis Konstantoulakis, Ioannis Sperelakis, George Kontakis, Nikolaos Tzanakis, Rozalia Dimitriou

**Affiliations:** 1 Medical School, University of Crete, Heraklion, GRC; 2 Orthopaedics, General Hospital of Chania "Agios Georgios", Chania, GRC; 3 Orthopaedics and Traumatology, University Hospital of Heraklion, Heraklion, GRC; 4 Orthopaedic Surgery, University Hospital of Heraklion, Heraklion, GRC; 5 Thoracic Medicine, University Hospital of Heraklion, Heraklion, GRC; 6 Orthopaedics, University Hospital of Heraklion, Heraklion, GRC

**Keywords:** crete, epidemiology, greece, legg-calvé-perthes disease, low incidence, population-based study

## Abstract

Background: Legg-Calvé-Perthes disease (LCPD) is an uncommon pediatric osteonecrosis of the femoral head with substantial geographic variation in reported incidence. This study estimated the population-based incidence of LCPD in Crete (Greece) and contextualized findings through a narrative review of contemporary international literature.

Methods: We conducted a population-based prospective study of children aged 4-16 years in Crete between 2021 and 2024. Disease incidence was calculated using age-specific annual population counts and expressed per 100,000 child-years at risk. Annual population denominators were obtained from the Hellenic Statistical Authority. Incidence rates (per 100,000 child-years) and their 95% confidence intervals (CIs) were calculated assuming a Poisson distribution. In parallel, a narrative review (January 2015-September 2025) was conducted in PubMed/MEDLINE and Ovid to identify population-based studies reporting LCPD incidence or epidemiology following predefined search and selection criteria.

Results: Over the four-year study period, a total of 401,900 child-years at risk were recorded, during which a total of 10 children were diagnosed with LCPD in Crete (11 affected hips; one bilateral case), yielding an overall hip-based incidence of 2.74 per 100,000 child-years (95% CI: 1.37-4.88). Annual incidence was 4.83 per 100,000 child-years in 2021 and approximately 2.00 per 100,000 child-years in 2022-2024. The mean age at presentation was 6.50 years, with a male predominance. The literature review identified eight eligible studies demonstrating marked international heterogeneity in incidence (0.2-29.2 per 100,000) and consistent male predominance.

Conclusions: LCPD incidence in Crete (2021-2024) was low and within the lower-to-mid range of international estimates. Findings reflect a population-based setting but should be interpreted cautiously due to limited case numbers and the absence of a control group.

## Introduction

Legg-Calvé-Perthes disease (LCPD) is an idiopathic osteonecrosis of the capital femoral epiphysis occurring in childhood, with a pathogenesis that remains incompletely understood [1]. The prevailing model involves transient impairment of femoral head perfusion, leading to epiphyseal ischaemia and subsequent osteonecrosis, potentially influenced by mechanical loading during skeletal growth. Several systemic, environmental, and genetic factors have also been implicated, including low gestational age and birth weight, maternal smoking during pregnancy, congenital malformations, coagulation abnormalities, and neurodevelopmental disorders, whereas the role of overweight and obesity remains less clearly established [1,2].

Radiographic assessment remains central to staging, prognostication, and treatment planning in LCPD. The Waldenström classification describes the temporal progression of the disease, while the Catterall and Herring lateral pillar classifications assess the extent and severity of epiphyseal involvement, particularly during the fragmentation stage, thereby providing prognostic information relevant to clinical decision-making [3,4].

The incidence of LCPD demonstrates marked geographic variability, with reported rates ranging from 0.2 to 29.0 per 100,000 children aged 0-14 years. The disease typically presents between four and eight years of age and shows a strong male predominance, commonly estimated at approximately 5:1 [2,5]. This epidemiological heterogeneity may reflect differences in study design, age denominators, population structure, case ascertainment, and environmental or genetic background. Although prevalence data among adolescent males in South Korea have reached approximately 71 per 100,000, these estimates are not directly comparable to incidence rates but further highlight population-level variability [6].

Despite the extensive international literature, no population-based study has previously reported the incidence of LCPD in Greece. This lack of national or regional epidemiological data represents an important knowledge gap, particularly given the marked geographic variability reported across other populations. Accordingly, the primary objective of this study was to estimate the hip-based incidence of LCPD among children aged 4-16 years in Crete, Greece, during the period 2021-2024, using person-time methodology and child-years at risk. The secondary objective was to conduct a focused narrative review of contemporary population-based incidence studies published between 2015 and 2025, in order to contextualize the Cretan findings within the international epidemiological literature and assess how they compare with reported incidence patterns across different populations.

## Materials and methods

Study design and setting

We conducted a prospective population-based incidence study in Crete, Greece, to estimate the annual and cumulative hip-based incidence of LCPD during the period from January 1, 2021, to December 31, 2024. Crete represents a geographically defined island population, with pediatric orthopedic care delivered predominantly through two main referral centers by three pediatric orthopedic surgeons. These services constitute the principal diagnostic and referral pathways for suspected Perthes disease on the island. Therefore, case ascertainment was expected to be close to complete, although mild or atypical cases managed exclusively outside these pathways may not have been captured. Year-specific population denominators for children aged 4-16 years were obtained from the Hellenic Statistical Authority.

Participants and eligibility criteria

The study population included children aged 4-16 years residing in Crete during the study period. Eligibility was defined by age, residence within the region, and a new diagnosis of LCPD during the study period. As the at-risk population was age-defined and changed over time, the study population was considered dynamic, with children entering and leaving the denominator as they moved into or out of the specified age range. Incident cases were identified prospectively during routine clinical care at the participating referral centers. Children diagnosed before January 1, 2021, children residing outside Crete, and children outside the predefined age range were excluded.

Case ascertainment and data collection

All newly diagnosed cases of LCPD during the study period were recorded prospectively by the participating pediatric orthopedic surgeons. Case identification was based on clinical presentation compatible with LCPD, including limping, hip or groin pain, restricted hip range of motion, or referred knee pain, together with radiographic confirmation of osteonecrosis and/or fragmentation of the capital femoral epiphysis during routine diagnostic assessment. Cases were identified through the participating referral centers, which represent the principal pediatric orthopedic pathways for suspected Perthes disease in Crete. No regional LCPD registry exists; therefore, case ascertainment relied on prospective recording of newly diagnosed cases by the treating pediatric orthopedic surgeons. Although this pathway was expected to capture the majority of clinically diagnosed cases on the island, mild or atypical cases managed exclusively outside these referral pathways may not have been identified.

For each patient, demographic and clinical information was collected, including sex, age at presentation, laterality, birth weight, weight percentile, socioeconomic status, radiographic classification, selected comorbidities, exposure to smoking, participation in organized athletic activities, and selected laboratory or clinical variables considered relevant to previously reported risk factors. These included coagulation abnormalities, thyroid dysfunction, vitamin D deficiency, and neurodevelopmental disorders.

Overweight and obesity were defined according to age- and sex-adjusted weight percentiles. Low birth weight was defined as birth weight <2,500 g. Vitamin D deficiency, thyroid dysfunction, coagulation abnormalities, and neurodevelopmental disorders were recorded when documented in the medical record or laboratory evaluation. Socioeconomic status and smoking exposure were recorded descriptively based on available clinical documentation.

Outcome definition and unit of analysis

The primary outcome was the incidence of newly diagnosed LCPD in the pediatric population of Crete during the study period. Patient-level analyses were used for demographic and clinical characteristics. For incidence estimation, affected hips were used as the predefined numerator. Therefore, because one child presented with bilateral disease, the cohort comprised 10 children and 11 affected hips, and the incidence was reported as hip-based incidence per 100,000 child-years. This distinction between patient-level descriptive analyses and hip-based incidence estimation was applied consistently throughout the manuscript.

Statistical analysis

Incidence was estimated using person-time methodology to account for the dynamic nature of the age-defined pediatric population. Annual age-specific population counts were summed across the study period to calculate the total number of child-years at risk. Incidence was calculated as the number of newly diagnosed affected hips divided by the total number of child-years at risk and expressed per 100,000 child-years. Annual and cumulative incidence rates were calculated using the same predefined hip-based numerator. Confidence intervals (CIs) for incidence rates were calculated using a Poisson approach, as appropriate for rare events and small case numbers. Rate calculations and 95% CIs were performed using the MedCalc online statistical calculator for comparison of rates. Descriptive statistics were used to summarize demographic and clinical characteristics of the identified cases. Continuous variables are presented as means, and categorical variables as counts and percentages. Available-case analysis was used for descriptive variables; no imputation was performed because of the small sample size and descriptive nature of the study.

Literature review

To contextualize the findings, we conducted a focused narrative review of contemporary studies reporting population-based incidence data for LCPD. PubMed/MEDLINE and Ovid were searched for articles published between January 2015 and September 2025. The search strategy included combinations of the following terms: “Legg-Calvé-Perthes disease,” “Perthes disease,” “Legg Perthes,” “incidence,” “epidemiology,” “population-based,” “children,” and “adolescents.” Reference lists of relevant articles were also screened manually to identify additional eligible studies.

Studies were included if they reported population-based incidence data for LCPD in pediatric or adolescent populations and provided sufficient information on the study population, denominator, or incidence estimate. Studies were excluded if they focused primarily on surgical management, treatment outcomes, imaging findings without epidemiological data, non-population-based case series, adult populations, or conference abstracts. Titles and abstracts were screened first, followed by full-text assessment of potentially eligible articles. Eligible studies were then reviewed narratively, and key epidemiological data, including country or region, study period, age range, population denominator, number of cases, incidence estimates, and sex distribution, were extracted where available. Because this was a focused narrative review rather than a formal systematic review, no formal risk-of-bias assessment was performed, and no PRISMA flow diagram was generated.

Ethics

Official ethics approval for this study was granted by the Administrative Council of Heraklion University Hospital, following a positive recommendation from the Bioethics and Ethics Committee (Protocol No. 16/07-07-2021). The requirement for informed consent was waived because of the observational population-based design. Patient confidentiality was maintained throughout, and no directly identifiable personal information was included in the study database or manuscript.

## Results

Incidence and clinical characteristics of LCPD in Crete (2021-2024)

Between 2021 and 2024, 10 children were diagnosed with LCPD in Crete. Although 10 children were identified, the incidence was estimated using affected hips as the numerator; therefore, the child with bilateral disease contributed two incident cases, yielding a total of 11 affected hips. During the study period, 401,900 child-years at risk were recorded among children aged 4-16 years residing in Crete. The annual number of incident presentations was highest in 2021 and lower in the subsequent years. Using affected hips as the predefined numerator, the overall hip-based incidence was 2.74 per 100,000 child-years (95% CI: 1.37-4.88), with the highest annual incidence observed in 2021 and lower annual estimates during 2022-2024.

The mean age at presentation was 6.50 years, and boys accounted for 80% of cases. Among the nine children with unilateral disease, the right hip was affected in eight. Radiographic severity was most commonly classified as Herring C, followed by Herring B, while single cases were classified as Herring A and Herring B/C. Obesity above the 97th percentile was observed in 4 of 10 children, and low birth weight below 2,500 g was recorded in 4 of 10 children. Organized athletic activity was reported in 5 of 10 children, and low socioeconomic status predominated among cases for whom this information was available. No maternal smoking during pregnancy was reported, although parental smoking with possible secondhand smoke exposure was documented in 5 of 10 children (Table 1).

**Table 1:** Individual anthropometric and clinical characteristics of the 10 children with Legg-Calvé-Perthes disease. n/a: not applicable; F: female; M: male; ADHD: attention-deficit/hyperactivity disorder

Individual	Gender (F/M)	Presentation age (years)	Weight (percentile)	Coagulopathies	Other health problems	Thyroid dysfunction/vitamin D deficiency	ADHD, spectrum of autism	Athletic activities	Birth weight (grams)	Herring classification	Socioeconomic status	Affected side
1	M	7	>99^th^	n/a	n/a	Vitamin D deficiency	n/a	Yes	3200	C	Low	Left
2	F	9	>97^th^	Yes	n/a	n/a	n/a	n/a	2300	C	Low	Right
3	M	6	<25^th^	n/a	n/a	n/a	Yes	Yes	2120	B	Low	Right
4	M	7	<25^th^	n/a	n/a	n/a	n/a	Yes	3240	A	Low	Right
5	M	7	45^th^-55^th^	n/a	n/a	n/a	n/a	n/a	4000	C	Low	Right
6	M	4	>97^th ^	n/a	Yes (beta-thalassemia)	n/a	n/a	n/a	3700	B	Low	Right
7	M	4	30^th^-35^th^	n/a	n/a	n/a	n/a	Yes	2300	C	Low	Right
8	F	6	>97^th^	n/a	n/a	n/a	n/a	n/a	3100	B/C	n/a	Right
9	M	4	45^th^-55^th^	n/a	n/a	n/a	n/a	n/a	3150	C	n/a	Right
10	M	11	50^th^	n/a	Yes (open-heart surgery)	Yes (thyroid dysfunction)	n/a	Yes	1900	B	Low	Both

## Discussion

Incidence and demographics in LCPD studies

This study represents the first prospective population-based epidemiological investigation of LCPD in Greece. Our findings indicate that LCPD was uncommon in this Mediterranean island population during the 2021-2024 study period, with an overall hip-based incidence of 2.74 per 100,000 child-years among children aged 4-16 years. This estimate was calculated using person-years at risk, reflecting the dynamic nature of an age-defined pediatric population in which children enter and leave the denominator as they age into and out of the at-risk window. However, because the estimate is based on a small number of identified cases, it should be interpreted with appropriate caution and regarded as an initial population-based estimate rather than a definitive national incidence rate.

The focused narrative review identified eight contemporary population-based studies reporting on the epidemiology of LCPD worldwide. Across these studies, incidence estimates varied substantially, ranging from approximately 0.2 to 29.2 per 100,000 children or child-years [1,5]. This geographic variability may reflect differences in age ranges, population denominators, case definitions, diagnostic pathways, population structure, and case ascertainment methods. Within this context, the incidence observed in the present Cretan cohort lies toward the lower end of the internationally reported spectrum. Direct comparisons, however, should be interpreted cautiously because of methodological heterogeneity across studies. Mullan et al. also reported a declining incidence trend in Northern Ireland, further supporting the potential influence of temporal and population-specific factors on disease occurrence [7]. Although Kim et al. [6] reported prevalence rather than incidence among males in South Korea, this estimate was not included in the incidence range and is presented only as supplementary epidemiological context, because prevalence and incidence are distinct measures and are not directly comparable. The demographic profile of our cohort was generally consistent with previous reports. The mean age at presentation was 6.50 years, close to the age range most commonly described in the literature, and male predominance was observed. These findings align with prior population-based studies, in which LCPD most commonly presents during the early school years, and male-to-female ratios typically range between 3:1 and 5:1 [1,5]. Clinically, unilateral involvement predominated in our cohort, although one child presented with bilateral disease, resulting in 10 affected children and 11 affected hips.

Reported risk factors in LCPD studies

There remains no clear consensus regarding which risk factors play a primary role in the pathophysiology of LCPD, and existing studies have not evaluated a uniform set of exposures. In the present cohort, potential risk factors were assessed descriptively only. Given the small sample size and the absence of a control group, these observations should be interpreted as exploratory and hypothesis-generating rather than as evidence of association or causality.

Several investigations have reported associations between coagulation abnormalities and LCPD, including factor V Leiden mutations, elevated fibrinogen levels, and increased factor VIII activity [8-10]. Hedley et al. identified additional nonspecific coagulation defects, although they did not demonstrate a consistent association between factor V Leiden mutation status or fibrinogen concentrations and disease occurrence [11]. In contrast, Joseph et al. reported no significant relationship between thrombophilia and the development of LCPD [5]. This heterogeneity may reflect differences in sample size, patient selection, and the extent and timing of thrombophilia screening across studies. In the present cohort, only one child had a documented coagulation abnormality.

Low birth weight has also been proposed as a potential risk factor for LCPD and was observed in 4 of 10 children in our cohort. Bahmanyar et al. reported very low birth weight as an independent risk factor associated with an approximately 240% increase in LCPD risk [12]. A higher frequency of low birth weight among affected children has also been described by Loder and Skopelja and summarized by Hedley et al. [10,11]. Conversely, Joseph et al. found no significant association between birth weight and LCPD risk [5]. Given the small sample size of the present cohort, this observation should be interpreted cautiously and considered hypothesis-generating rather than confirmatory.

Parental smoking has been linked to LCPD in several studies. Joseph et al. identified multiple reports supporting this association, Johansson et al. demonstrated a specific association with maternal smoking, and Mullan et al. suggested that a reduction in maternal smoking may have contributed to declining incidence trends in Northern Ireland [5,13]. In our cohort, no maternal smoking during pregnancy was reported, although postnatal exposure to parental smoking was documented in 5 of 10 children. This distinction is important, as prenatal and postnatal smoking exposure may represent different biological and epidemiological pathways; however, no association can be inferred from the present descriptive data.

Other proposed risk factors include congenital malformations, neurodevelopmental disorders, and increased body weight. Hedley et al. reported an increased risk of LCPD among children with congenital malformations and neurodevelopmental disorders, including autism spectrum disorders and hyperkinetic conditions [11]. In our cohort, autism spectrum disorder was identified in one child. A total of 5 of 10 children were overweight or obese, consistent with some prior reports [6,7]; however, the remaining cases spanned lower weight percentiles. This distribution supports the concept of a multifactorial etiology, but does not allow causal interpretation in the absence of a control group and adequate statistical power.

Radiographic classification and clinical implications

A further limitation of the existing literature is the lack of uniformity in LCPD classification systems, which may reduce comparability across studies and contribute to heterogeneity in reported severity and outcomes. In the present study, disease severity was assessed using the Herring classification, with more than half of affected hips classified as stage B or higher. This finding should be interpreted descriptively, given the small cohort size, but it highlights the importance of maintaining a high index of clinical suspicion in children aged four to eight years presenting with limping or atypical pain patterns, including knee-referred pain.

Additional population-based studies from the United Kingdom and the United States have also reported incidence estimates within the lower international range [14,15]. The main epidemiological characteristics of the contemporary population-based studies included in the focused narrative review are summarized in Table 2.

**Table 2:** Population-based incidence or prevalence estimates, sex distribution, and age at diagnosis of Legg-Calvé-Perthes disease across published studies. * Kim et al. [6] reported prevalence among males in South Korea rather than incidence; therefore, this estimate was not included in the reported incidence range. n/a: not applicable

Authors/year	Country	Incidence (per 100000)	Males to females	Age (years) (mean/range)
Rodríguez-Olivas et al., 2022 [1]	Mexico	0.4-29.0	3-5:1	5-7
Joseph et al., 2023 [5]	UK, India, Norway	0.2-19.1	5:1	0-14
Kim et al., 2025 [6]*	South Korea	71.17 (only males)	Only males	n/a
Mullan et al., 2017 [7]	Northern Ireland	11.6 (1992-1998); 4.6 (2007-2013)	4.5:1 (1992-1998); 4:1 (2007-2013)	5.7 (1.8-12.4)
Hedley et al., 2025 [11]	Denmark	12.1	4.2:1	3-7
Johansson et al., 2017 [13]	Sweden	9.3	3.1:1	Males: 8; females: 9
Perry et al., 2022 [14]	UK	2.48	3:1	5.4
Kessler and Cannamela, 2018 [15]	USA (Southern California)	2.84	2:1 up to 6:1	4.7 ± 2.49

Strengths and limitations of this study

This study has several strengths. It represents the first prospective population-based incidence study of LCPD in Greece and provides contemporary epidemiological data from the 2021-2024 period. The use of person-time methodology and child-years at risk allowed incidence estimation in a dynamic pediatric population. In addition, Crete represents a geographically defined and demographically stable island population, with pediatric orthopedic care delivered predominantly through centralized referral pathways. This healthcare structure likely improved case capture and strengthened the internal validity of the incidence estimate.

Several limitations should also be acknowledged. The most important limitation is the small number of identified children with LCPD (n = 10), which resulted in limited statistical power, wide uncertainty around incidence estimates, and the inability to perform meaningful subgroup analyses. Therefore, clinical characteristics and potential risk factors in this cohort should be interpreted as descriptive and hypothesis-generating only, rather than as evidence of association or causality. The absence of a control group further precludes causal inference regarding potential risk factors.

Potential under-ascertainment should also be considered. Although the participating centers represent the principal pediatric orthopedic referral pathways for suspected Perthes disease in Crete, mild or atypical cases managed outside these pathways, including cases assessed in private clinics or not referred for specialist evaluation, may have been missed. In addition, no regional LCPD registry exists, and diagnostic variability across non-participating settings cannot be excluded.

The unit of analysis was an important methodological consideration. Demographic and clinical characteristics were analyzed at the patient level, whereas incidence was estimated using affected hips as the predefined numerator. Because one child had bilateral disease, the cohort comprised 10 children and 11 affected hips. This distinction has been clarified and applied consistently across the manuscript to improve transparency and reproducibility.

The focused narrative review also has limitations. Although predefined databases, search terms, eligibility criteria, and screening procedures were used, the review was not designed as a formal systematic review. Therefore, no formal risk-of-bias assessment was performed, and no PRISMA flow diagram was generated. Finally, some clinical variables, including socioeconomic status, smoking exposure, vitamin D deficiency, thyroid dysfunction, coagulation abnormalities, and neurodevelopmental disorders, were recorded according to available clinical documentation and medical records. Missing or incompletely documented variables were not imputed because of the small sample size and descriptive nature of the study.

Because the study was conducted in a geographically specific island population with centralized referral pathways, the findings may not be directly generalizable to larger, more heterogeneous populations or to healthcare systems with different referral structures. Nevertheless, the study provides an initial population-based estimate of LCPD incidence in Greece and may serve as a basis for future multicenter or national epidemiological studies.

## Conclusions

In conclusion, this study provides prospective, population-based data on the hip-based incidence of LCPD in Crete, a geographically defined island population with centralized pediatric orthopedic referral pathways. During the 2021-2024 study period, LCPD was uncommon, with an estimated incidence of 2.74 per 100,000 child-years among children aged 4-16 years. This estimate lies toward the lower end of the international range reported in population-based studies, although direct comparisons should be interpreted cautiously because of differences in study design, age denominators, diagnostic pathways, and case ascertainment methods. The findings should also be considered in light of the small number of identified cases, the possibility of under-ascertainment of mild or atypical presentations, and the absence of a control group, which precludes causal inference regarding potential risk factors and limits generalizability beyond similar populations. Further multicenter or national studies are needed to confirm these findings and better characterize the epidemiology of LCPD in Greece.
